# Items

**Published:** 1904-11

**Authors:** 


### ITEMS.

BATTLE & COMPANY, of Saint Louis, recently published Chart No. 3, of the series illustrating intestinal parasites. This plate depicts the tenia soluim—the pork-measle tapeworm and armed tapeworm.

THE DAVIS PHARMACEUTICAL COMPANY, of New York, presents an advertisement on page xi., setting forth the value of Guaisotol, to which attention is invited.

